# Impact of Sentinel Node Omission in Early-Stage Breast Cancers on Adjuvant Therapy Decision-Making

**DOI:** 10.1245/s10434-026-20145-x

**Published:** 2026-07-23

**Authors:** Jordan E Jackson, Sarah Hartman, Joshua Barkin, Hemi Thaker, Julia Chandler, Michael Alvarado

**Affiliations:** Helen Diller Comprehensive Breast Cancer Center, University of California, San Francisco, CA USA

## Abstract

**Background:**

Recent studies suggest that selective omission of sentinel lymph node biopsy (SLNB) in early-stage breast cancer may reduce morbidity without compromising oncologic outcomes. The impact of SLNB omission on eligibility for adjuvant therapies, including CDK4/6 inhibitors and partial breast irradiation (PBI), remains unclear, as both depend on nodal status.

**Methods:**

Using a single-institution registry (2012–2024), we identified women with cT1N0, ER+/HER2−, grade 1–2 breast cancer who would have been candidates for SLNB omission. Pathologic nodal status and eligibility for CDK4/6 inhibitors and PBI were assessed.

**Results:**

Among 1,194 patients, 130 (10.9%) had ≥1 positive sentinel lymph nodes, including five (0.4%) with ≥4 positive nodes. Nodal positivity decreased with age (18% <50 years vs. 7.4% ≥70 years (*p* < 0.001). Nodal positivity increased with tumor size (6.2% (cT1a), 6.6% (cT1b), 14.4% (cT1c); *p* < 0.001) and with tumor grade (8.5% (grade 1) vs. 12.4% (grade 2); *p* = 0.03). In women aged 50–59 years with cT1a/b tumors, nodal positivity did not differ by grade (6.8% grade 1 vs. 11.1% grade 2; *p* = 0.43). In women aged ≥60 years with cT1a/b tumors, rates were 3.1 and 2.1%, respectively (*p* = 1.00).

**Conclusions:**

Under current guidelines, SLNB omission would have excluded 18% of women <50 and 8.8% of women ≥50 from consideration for CDK4/6 inhibitors and PBI. Sentinel lymph node biopsy omission is unlikely to affect adjuvant treatment selection in carefully selected older women with small, low-grade tumors, particularly those aged ≥60 years with grade 1 cT1a/b disease, in whom occult nodal positivity was only 2.1%. In contrast, younger patients, those with cT1c tumors, and those with higher-grade disease demonstrated substantially higher rates of nodal involvement, suggesting that SLNB continues to provide clinically actionable information that may influence eligibility for CDK4/6 inhibitors and partial breast irradiation. These findings support a risk-adapted rather than universal approach to SLNB omission.

The management of the axilla in early-stage breast cancer has undergone a profound shift from mandatory surgical staging toward a strategy of selective de-escalation. The transition from axillary lymph node dissection to sentinel lymph node biopsy (SLNB) was the first major de-escalation, and recent landmark trials, such as SOUND^1^ and INSEMA,^2^ have provided a clinical foundation for further de-escalating to omitting SLNB. In these studies of patients with small (cT1) and clinically node-negative (cN0) tumors, the complete omission of SLNB did not compromise oncologic outcomes.^1,2^

However, this evolution introduces an important paradox. As surgical staging is reduced, the clinical importance of nodal information in guiding adjuvant therapy has increased. Contemporary treatment strategies increasingly rely on pathologic nodal status to inform both systemic therapy selection and radiation planning. 

The desire to reduce surgical morbidity may inadvertently conflict with the ability to provide optimized adjuvant care. The NATALEE and MonarchE trials illustrate this shift as nodal status is a key determinant of eligibility.^3,4^ In the NATALEE trial, patients with hormone receptor (HR)-positive, HER2-negative early breast cancer (stage II–III) were randomized to receive standard endocrine therapy with or without ribociclib. The addition of ribociclib was associated with improved invasive disease-free survival.^3^ In MonarchE, patients with HR positive, HER2-negative, high-risk early breast cancer were randomized to receive standard endocrine therapy for at least 5 year with or without abemaciclib and the addition of abemaciclib significantly improved invasive disease-free survival, distant recurrence-free survival, and overall survival.^5^ High-risk disease was defined as either 1) ≥4 positive axillary lymph nodes (ALN), or 2) 1–3 positive ALN with Grade 3 histology and/or tumor size ≥5 cm.^4^ As a result, omission of SLNB may preclude access to therapies associated with improved invasive disease-free and overall survival.

Similarly, nodal staging plays a central role in radiation decision-making. Partial breast irradiation (PBI) offers a significantly de-escalated treatment schedule that improves patient quality of life and reduces the overall healthcare burden.^6^ However, current guidelines generally require pathologically confirmed node-negative (pN0) status.^7^ In the absence of an SLNB, patients may be defaulted to a more intensive and burdensome course of whole breast irradiation (WBI) due to staging uncertainty rather than true clinical necessity.

As institutions work to establish formal protocols for the omission of SLNB in early-stage breast cancer, it is imperative to quantify the multidisciplinary trade-offs involved. This study aimed to quantify the proportion of patients who would lose eligibility for adjuvant systemic therapy or radiation de-escalation if SLNB were omitted and to identify clinical subgroups in whom this trade-off is minimized.

## Methods

### Study Design

Using a single-institution cancer registry, we identified patients diagnosed with clinical stage T1N0, estrogen receptor–positive, HER2-negative, grade 1 or 2 invasive breast cancer between 2012 and 2024. We sought to determine how many of these early-stage patients would lose eligibility for adjuvant CDK 4/6 inhibitors or PBI if SLNB were omitted.

Data collected from the registry included patient age at diagnosis, histologic grade from final operative pathology, and clinical T-stage subclassification. Pathologic nodal status was analyzed following surgical intervention. Sentinel lymph nodes were defined as hot (technetium Tc99m sulfur colloid or MagTrace), blue (isosulfan blue or methylene blue dye), or a combination when dual-tracer mapping was utilized.

### Eligibility Criteria

*CDK4/6 inhibitor therapy.*Eligibility for adjuvant CDK4/6 inhibitor therapy was defined according to the NATALEE and MonarchE trial criteria.^3,4^ This includes patients with hormone receptor–positive, HER2-negative early breast cancer at risk of recurrence, including those with 4 positive lymph nodes or select high-risk disease with 1–3 positive nodes. For the purposes of this study, we focused specifically on patients whose eligibility would be contingent on the identification of pathologic nodal involvement, as these individuals would be most directly impacted by omission of sentinel lymph node biopsy.

*Partial breast irradiation. *Eligibility for PBI was defined according to the IMPORT LOW trial protocol: age ≥50 years, unifocal invasive ductal carcinoma, ER-positive tumors, grade 1–3, tumor size ≤3 cm, node-negative (pN0), and surgical margins ≥2 mm.^8^ The American Society for Radiation Oncology (ASTRO) guidelines support these patients as suitable for PBI.^9^

### Statistical Analysis

Pathologic nodal status and resulting eligibility for adjuvant PBI and CDK4/6 inhibitor therapy were evaluated in accordance with the trial-specific criteria described above. Descriptive statistics were used to summarize patient demographics and clinicopathologic characteristics; continuous variables were reported as medians, and categorical variables as frequencies with percentages.

Chi-squared tests were used to assess associations between categorical clinicopathologic variables, including age group, histologic grade, and clinical T-stage subclassification, and the likelihood of pathologically node-positive disease. Cochran–Armitage trend tests were performed to evaluate for ordered trends in nodal positivity across incremental categories of age, grade, and *T* stage. Multivariable logistic regression was then used to identify independent predictors of node-positive disease, with results reported as odds ratios with 95% confidence intervals.

The proportion of patients who would have lost eligibility for adjuvant CDK4/6 inhibitor therapy or PBI based solely on pathologic nodal status was calculated and stratified by clinicopathologic subgroup. All statistical tests were two-sided, with a significance threshold of *p* ≤ 0.05.

## Results

A total of 1,194 clinically node negative patients met the specified inclusion criteria (Table 1). Overall, 130 patients (10.9%) were found to have at least one positive sentinel lymph node on final pathologic review (Table 2). Of those, 125 (10.5%) had 1–3 positive nodes and five (0.4%) had ≥4 positive lymph nodes.

**Table 1:** Patient and tumor characteristics

Category	n	%
Age		
<50 yrs	272/1194	22.8
50–59 yrs	286/1194	24.0
60−69 yrs	325/1194	27.2
≥70 yrs	311/1194	26.0
Grade		
Grade 1	461/1194	38.6
Grade 2	733/1194	61.4
T Stage		
cT1mi	56/1194	4.7
cT1a	119/1194	10.0
cT1b	395/1194	33.0
cT1c	624/1194	52.3

**Table 2:** Nodal positivity rates in select groups

Category		n	%	*p*-value
≥1 Positive sentinel lymph node		130	10.9	
≥4 Positive sentinel lymph node		5	0.42	
Rate of nodal positivity by age group				
	<50 yrs	49/272	18.0	*P*<0.001
	50–59 yrs	32/286	11.2	
	60–69 yrs	26/325	8.0	
	≥70 yrs	23/311	7.4	
Rate of nodal positivity by tumor grade				
	Grade 1	39/461	8.5	*P*=0.0.3
	Grade 2	91/733	12.4	
Rate of nodal positivity by clinical tumor size				
	cT1a	8/119	6.7	*P*<0.001
	cT1b	26/395	6.6	
	cT1c	90/624	14.4	
Nodal positivity among patients 50-59 years with tumors ≤1 cm (cT1a and cT1b)				
	Grade 1	4/59	6.8	*P*=0.53
	Grade 2	7/63	11.1	
Nodal positivity among patients ≥ 60 years with tumors ≤1 cm (cT1a and cT1b)				
	Grade 1	3/114	2.1	*P*=1
	Grade 2	5/1614	3.1	

### Nodal Positivity by Age

**Fig. 1:**
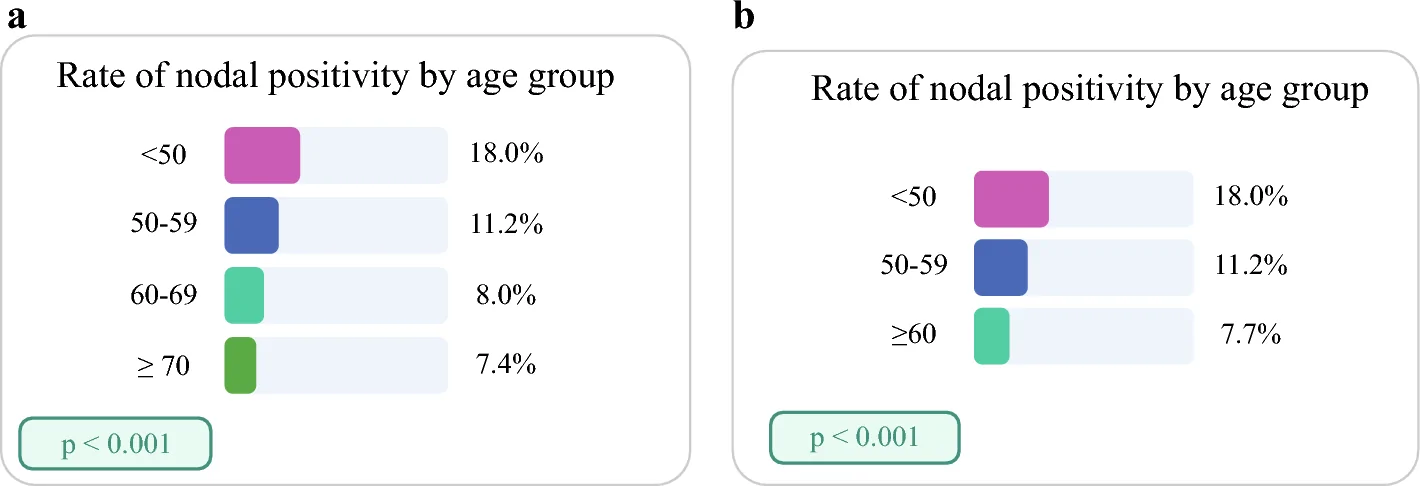
Nodal positivity rate stratified by age group

Nodal positivity was significantly associated with younger age. Women younger than 50 years were twice as likely to be node positive compared with those older than 50 years (18% vs. 8.8%; odds ratio [OR] 2.28, 95% confidence interval [CI] 1.54–3.36, *p* < 0.001). When further stratified by age, the frequency of nodal involvement demonstrated a statistically significant stepwise decline: 18% in women under 50 years; 11.2% in women aged 50–59; 8% in those aged 60–69; and 7.4% in those aged 70 and older (Fig. 1a *p*< 0.001). There was no statistically significant difference in nodal positivity between patients aged 60–69 and those aged 70 and older. Based on this similarity, these groups were combined into a single cohort aged 60 years and older, with a nodal positivity rate of 7.7%. In this combined analysis, the association between age group and nodal positivity remained statistically significant (Fig. 1b *p*<0.001).

### Nodal Positivity by Tumor Grade and T Stage

**Fig. 2:**
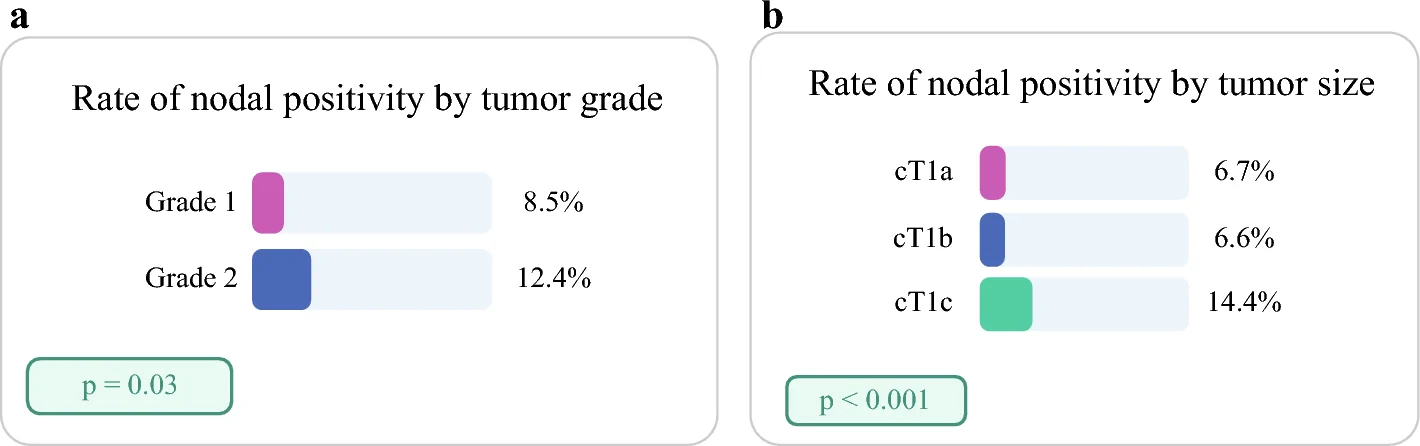
Nodal positivity rate by (**a**) tumor grade and (**b**) T stage

Tumor biology and size also served as strong indicators of nodal status (Fig. 2). Overall, the proportion of node positivity was significantly higher in patients with grade 2 tumors (*n* = 91/733, 12.4%) compared with those with grade 1 tumors (*n* = 39/461, 8.5%; *p* = 0.030; Fig. 2a). We then further stratified by age using 50 years as a marker for menopausal status. Among those older than 50 years, the proportion of node-positive cases was significantly higher in grade 2 (*n* = 62/569, 10.9%) than in grade 1 (*n* = 19/353, 5.4%; *p* = 0.0019), whereas no difference was observed among premenopausal patients (grade 2: *n* = 29/164, 17.7% vs. grade 1: *n* = 20/108, 18.5%; *p* = 0.87).

Node positivity also differed by tumor size (cT1a: *n* = 8/130, 6.2%; cT1b: *n* = 26/395, 6.6%; cT1c: *n* = 90/624, 14.4%; *p* = 0.0011), with significantly higher rates in cT1c compared with both cT1a (*p* = 0.005) and cT1b (*p* < 0.001), whereas no difference was observed between cT1a and cT1b (Fig. 2b *p*= 0.88).

### Combined Subgroup Analysis

**Fig. 3:**
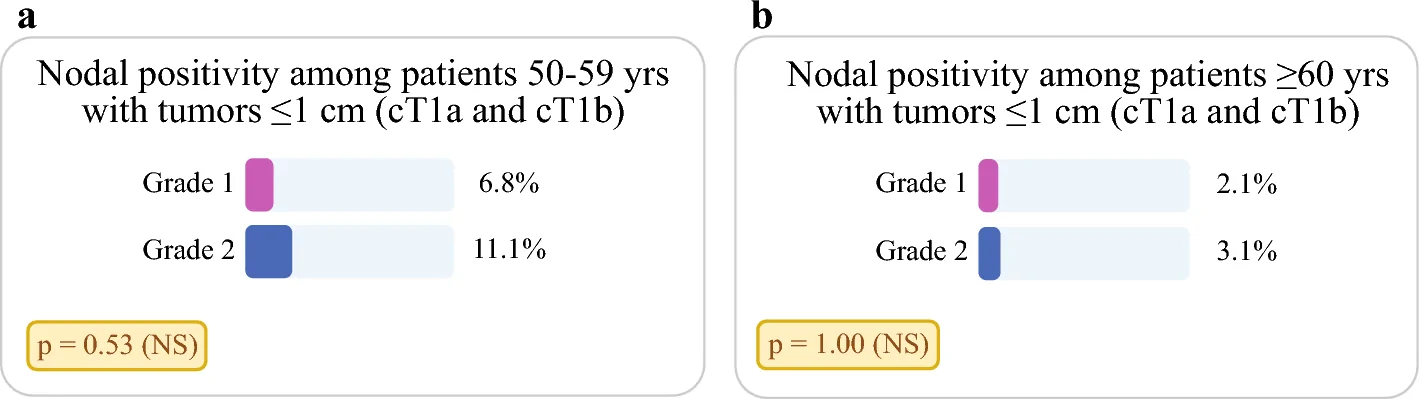
Subgroup Analysis showing (**a**) nodal positivity among patients age 50–59 years with tumors £1 cm and (**b**) nodal positivity among patients ^3^60 years with tumors £1 cm

By excluding factors we found to be associated with an increased risk for nodal positivity (age <50, tumor size >1 cm, grade 2), we sought to identify low-risk subgroups that could warrant SLNB omission. Among women aged 50–59 with grade 1, cT1a or cT1b tumors, we found that the nodal positivity rate was 6.8% (Fig. 3a). Nodal positivity was further reduced among older patients: women aged ≥60 years with cT1a or cT1b and grade 1 tumors demonstrated a nodal involvement rate of just 2.1% (Fig. 3b).

### Overall Analysis

Based on final pathologic nodal status, omission of SLNB would have resulted in loss of nodal information relevant to adjuvant treatment selection in 130 of 1,194 patients (10.9%). Among these patients, 125 (10.5%) had 1–3 positive lymph nodes and five (0.4%) had ≥4 positive lymph nodes, meeting nodal burden criteria incorporated into contemporary CDK4/6 inhibitor eligibility algorithms. Rates of occult nodal positivity varied by subgroup, including 18% in women <50 years, 14.4% in cT1c tumors, and 2.1% in women ≥60 years with grade 1 cT1a/b tumors. Conversely, 1,064 of 1,194 patients (89.1%) were pathologically node-negative and would have lacked pathologic confirmation of pN0 status for PBI eligibility determination if SLNB had been omitted.

## Discussion

This study highlights a central tension in contemporary breast cancer care with surgical management of the axilla being de-escalated and the clinical value of nodal information continuing to expand. In our cohort, 10.9% of clinically node-negative patients harbored occult nodal metastases.

With respect to adjuvant systemic therapy, only a small fraction of patients demonstrated high nodal burden, thus meeting traditional high-risk criteria for CDK4/6 inhibitor therapy independent of other features. However, the majority of node-positive patients would fall into an intermediate-risk category in which nodal status contributes significantly to treatment selection. For radiation therapy, omission of SLNB would preclude confirmation of pathologic node-negative (pN0) status in all patients. As a result, patients who would otherwise meet clinicopathologic criteria for PBI may instead be directed toward whole breast irradiation due to uncertainty in nodal staging. These findings suggest that omission of SLNB does not simply reduce surgical morbidity, but it also may remove clinically actionable information in a meaningful subset of patients.

Emerging real-world and institutional analyses have demonstrated that only a minority of patients with limited sentinel lymph node involvement are subsequently upstaged to ≥4 positive nodes after completion axillary dissection, the key threshold for high-risk classification in MonarchE. Recent population-based studies similarly suggest that the probability of identifying additional nodal disease sufficient to alter CDK4/6 eligibility is low in patients with 1–2 positive sentinel nodes and no other high-risk features, indicating that SLNB may infrequently change systemic therapy recommendations.^10,11^

An important consideration is that the decision to omit SLNB is based on preoperative clinical and biopsy findings, whereas decisions regarding adjuvant therapy are ultimately informed by final surgical pathology. Final pathology may occasionally identify higher-risk features, such as larger tumor size, grade upgrade, or lymphovascular invasion, which were not evident on core biopsy. This consideration is particularly relevant when applying risk-adapted omission strategies based on tumor size and grade, as these characteristics may differ between biopsy and final pathology. In such circumstances, patients may undergo multidisciplinary reassessment, and additional axillary staging may be considered in selected cases to better inform adjuvant systemic therapy and radiation recommendations. This highlights the iterative nature of multidisciplinary treatment planning and underscores that risk assessment continues to evolve throughout the course of care.

### Implications for Women Younger than 50

The impact of SLNB omission is most pronounced in the premenopausal population. In our cohort, 18% of women younger than 50 were node-positive at the time of SLNB. Under current treatment paradigms informed by the NATALEE and MonarchE trials,^3,4^ nodal positivity is a key determinant of eligibility for adjuvant CDK4/6 inhibition. Omission of SLNB in this population therefore introduces a significant risk of systemic under-treatment. Given the significant improvement in invasive disease-free survival demonstrated with ribociclib and abemaciclib in high-risk early breast cancer, the 18% rate of occult nodal involvement observed in our younger patients suggests that SLNB remains essential for appropriate systemic therapy selection in patients younger than 50 years. These findings align with subgroup analyses from MONARCH-E and NATALEE, which demonstrate that the absolute benefit of CDK4/6 inhibition is enriched in node-positive and higher-risk younger patients, reinforcing the role of nodal status as a key determinant of adjuvant treatment escalation in premenopausal populations.^3,4^

In addition, radiation de-escalation may be inadvertently limited for patients younger than 50. Current guidelines for PBI require pathologically confirmed node-negative status.^7^ Without SLNB, nodal status remains unknown, and patients may be excluded from PBI despite being appropriate candidates. In our cohort, the majority of patients younger than 50 were node-negative; thus, SLNB omission risks denying a substantial proportion access to de-escalated radiation therapy due to staging uncertainty rather than true ineligibility.

### Opportunities for De-escalation in Women Age 50 and Older

In contrast, our data suggests a much clearer path for de-escalation in women ≥50 with favorable tumor biology, particularly in those age ≥60. Among patients aged 60 and older with grade 1, cT1a or cT1b tumors, the nodal positivity rate was just 2.1%. In this population, the likelihood that SLNB findings would alter eligibility for adjuvant systemic therapy or radiation planning is exceedingly low. These findings support omission of SLNB as a high-value strategy in carefully selected older patients, preserving multidisciplinary treatment options for the vast majority while avoiding surgical morbidity.

### Role of Tumor Size

Tumor size emerged as an additional important discriminator of nodal risk. Among postmenopausal women, nodal positivity increased from 4.6% in cT1b tumors to 12.8% in cT1c tumors, suggesting a clinically meaningful threshold at 1 cm. This finding supports a more cautious approach to SLNB omission in T1c disease, where omission may result in loss of actionable staging information as more than 12% of these patients would lose access to indicated adjuvant CDK4/6 inhibitors or PBI.

### Limitations

This study has several important limitations. First, the retrospective, single-institution design limits generalizability and introduces potential selection bias. Practice patterns, patient demographics, and referral patterns at our institution may differ from those at community hospitals or other academic centers, and our findings may not be applicable to all clinical settings. In addition, the subgroup analyses used post-hoc stratification based on variables identified within the dataset as associated with nodal positivity, which may introduce exploratory bias and limits the ability to draw definitive causal inferences from these subgroup findings. Second, as with all cancer registry studies, we are limited by the completeness and accuracy of the data abstracted from medical records. Third, our study lacks long-term oncologic outcomes data, including recurrence, disease-specific survival, and overall survival. Without these endpoints, we cannot assess whether the occult nodal disease detected by SLNB in our cohort ultimately influenced clinical outcomes or whether omission of SLNB in low-risk subgroups would have resulted in worse oncologic results. Fourth, eligibility criteria for CDK4/6 inhibitor therapy and PBI continue to evolve, and the specific trial criteria used in this analysis (NATALEE, MonarchE, and IMPORT LOW) may not reflect current or future practice standards. Finally, our study period spanned 2012–2024, during which time both surgical practice patterns and adjuvant treatment paradigms changed substantially. This may have introduced unmeasured confounding and limits the applicability of our findings to contemporary practice.

## Conclusions

Our findings support a selective, risk-adapted approach to SLNB omission rather than broad application across all patients with clinically node-negative, hormone receptor-positive breast cancer. Women younger than 50 years, those with cT1c tumors, and those with higher-grade disease demonstrated the highest rates of occult nodal involvement and are therefore most likely to lose clinically actionable staging information that may influence eligibility for adjuvant CDK4/6 inhibitors and partial breast irradiation. Conversely, women aged ≥60 years with grade 1, cT1a/b tumors had a nodal positivity rate of only 2.1%, identifying a subgroup in whom SLNB omission may be reasonable with minimal impact on multidisciplinary treatment planning. These data suggest that decisions regarding SLNB omission should incorporate patient age, tumor size, and tumor biology and should be made within a multidisciplinary framework that balances the benefits of surgical de-escalation against the potential loss of information needed for contemporary adjuvant therapy selection. Future prospective studies are needed to validate these risk thresholds and to assess patient-reported outcomes and long-term oncologic safety in cohorts managed without SLNB.
